# Supplementary material for: Recovery of Dynamics and Function in Spiking Neural Networks with Closed-Loop Control
Source: PLoS Comput Biol. 2016 Feb 1;12(2):e1004720. doi: 10.1371/journal.pcbi.1004720 (PMC4734620; doi:10.1371/journal.pcbi.1004720)
Supplement: S1 Text — (PDF) [file pcbi.1004720.s001.pdf]

Effects of neural heterogeneity: bursting neurons

We test how well DFC performs under neural heterogeneity by substituting a fraction of regular spiking neurons with bursting ones. This particular choice is motivated by the fact that around 30% of neurons in the GPI/STN ([1],H. Bergman: personal communication) exhibit bursting activity. We used a novel implementation of a bursting neuron type, in which the f-I curve of the neuron is not affected by the generation of bursting spike patterns (Saharasnamam, Vlachos, Aertsen, Kumar, under revision). It is evident that the existence of bursting neurons does not decrease the efficacy of the controller, which upon activation sufficiently suppresses oscillations (**S3 Fig**). These results suggest that DFC is effective in a wide range of networks where single neuron properties may be of secondary importance. Indeed theoretical work on the control of time-delayed system suggests that DFC is applicable to any system that undergoes a supercritical Hopf bifurcation and it may work in other types of bifurcations as well [2, 3]. Thus, for the application of DFC on the mean-field of the network activity neuronal heterogeneity does not pose a serious problem, which renders DFC particularly relevant for neuroprosthetic applications.

References

[1] Tachibana Y, Iwamuro H, Kita H, Takada M, Nambu A. Subthalamo-pallidal interactions underlying parkinsonian neuronal oscillations in the primate basal ganglia. Eur J Neurosci. 2011;34(9):1470–1484.

[2] Pyragiene T, Pyragas K. Delayed feedback control of forced self-sustained oscillations. Phys Rev E - Stat Nonlinear, Soft Matter Phys. 2005;72(2):1–9.

[3] Pyragas K. A Twenty-Year Review of Time-Delay Feedback Control and Recent Developments. IEICE Proceeding Ser. 2014;1:683–686.
