# Supplementary material for: Recovery of Dynamics and Function in Spiking Neural Networks with Closed-Loop Control
Source: PLoS Comput Biol. 2016 Feb 1;12(2):e1004720. doi: 10.1371/journal.pcbi.1004720 (PMC4734620; doi:10.1371/journal.pcbi.1004720)
Supplement: S1 Table — (PDF) [file pcbi.1004720.s002.pdf]

**Neuron Parameters**		
Spike threshold	u_{th}	20 mV
Reset potential	u_r	14 mV
Resting potential	u_{rest}	0 mV
Refractory period	t_{ref}	1 ms
Membrane capacitance	C_m	250 pF
Membrane time constant	τ_m	10 ms
**Synaptic Parameters**		
Synaptic rise time	τ_r	1 ms
Synaptic decay time	τ_d	1 ms
Synaptic weight	J	
Synaptic delay	d	2 ms
**Input Parameters**		
Inhibitory network		
Input, mean - I population	μ_I	14 mV
Input, variance - I population	σ_I	6 mV
Excitatory-Inhibitory network		
Input, mean - E population	μ_E	20.8 mV
Input, mean - I population	μ_I	16.5 mV
Input, variance - E population	σ_E	20.8 mV
Input, variance - I population	σ_I	16.5 mV
**Network Parameters**		
Inhibitory network		
Number of inhibitory neurons	N_I	$10,000$
Excitatory-Inhibitory network		
Number of excitatory neurons	N_E	$8,000$
Number of inhibitory neurons	N_I	$2,000$
**Control Parameters**		
Inhibitory network		
Control input	I_K	$0-300$ mV
Control delay, direct control	d_c	$0-30$ ms
Control delay, differential control	d_{c1},d_{c2}	$0-30$ ms, 1 ms
Excitatory-Inhibitory network		2
Control gain	K	$0-300$ mV
Control delay, direct control	d_c	$0-30$ ms
Control delay, differential control	d_{c1},d_{c2}	$0-30$ ms, 1 ms
