# Supplementary figures and images for: Recovery of Dynamics and Function in Spiking Neural Networks with Closed-Loop Control
Source: PLoS Comput Biol. 2016 Feb 1;12(2):e1004720. doi: 10.1371/journal.pcbi.1004720 (PMC4734620; doi:10.1371/journal.pcbi.1004720)

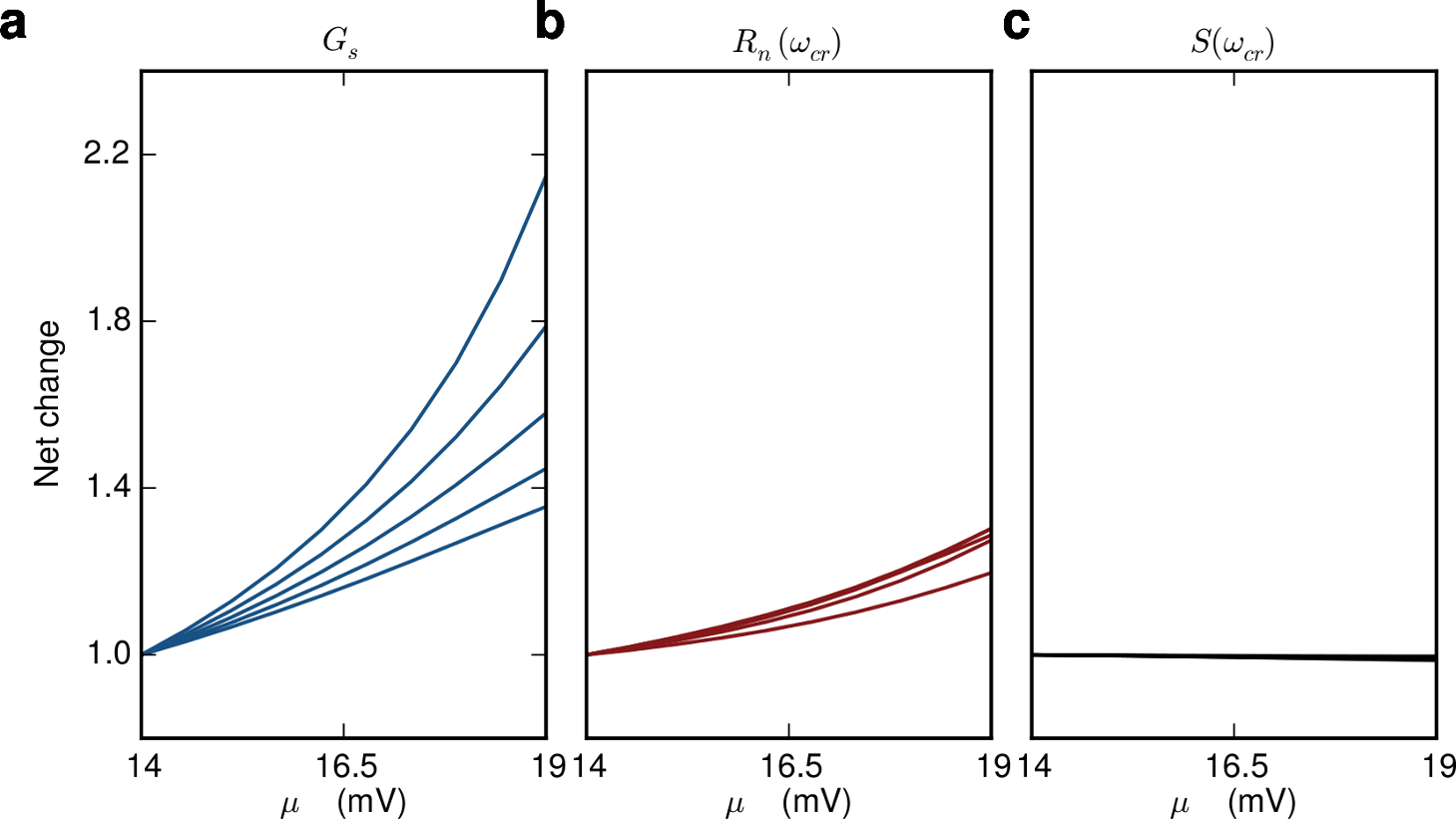

Supplement: S1 Fig — In order to maintain constant firing rates in each population we increase the mean input μ while decreasing the variance of the input σ. (a) Moving in the state-space while maintaining the firing-rates yields significant changes in the static gain G s. (b) The changes in the normalized neuronal response R n are modest (b) and the changes in the normalized synaptic response S n are negligible (c). (TIF) [file pcbi.1004720.s003.tif]

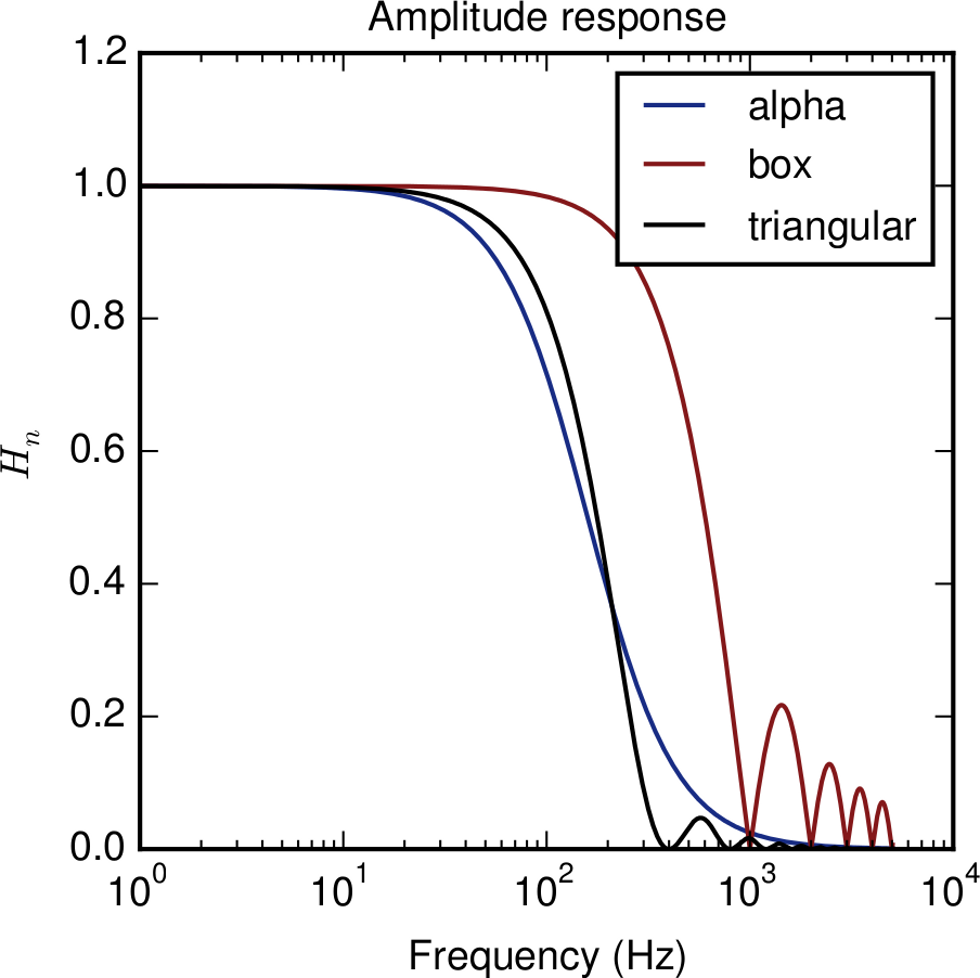

Supplement: S2 Fig — S n(ω) Various control kernels have very similar amplitude responses for the relevant frequency range f<100 Hz. (TIF) [file pcbi.1004720.s004.tif]

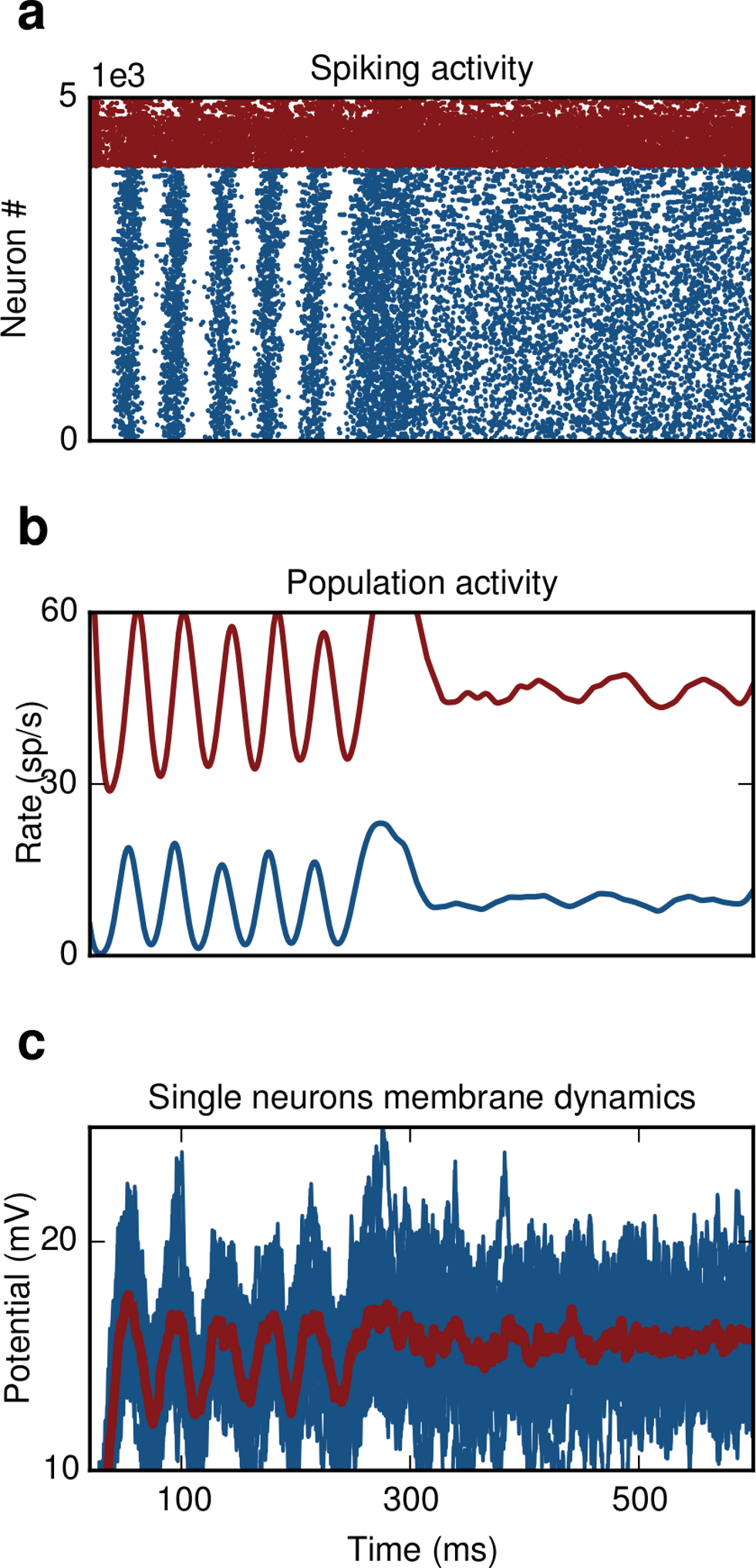

Supplement: S3 Fig — (a) Raster plot. Replacing regular spiking by bursting neurons (top 30% in excitatory and inhibitory population) does not compromise the effects of control. (b) Population activity of E-neurons (blue) and I-neurons (red). (c) Single membrane potential trajectories of ten randomly chosen E-neurons in the network. The averaged trace of the subthreshold dynamics is shown in red. (TIF) [file pcbi.1004720.s005.tif]

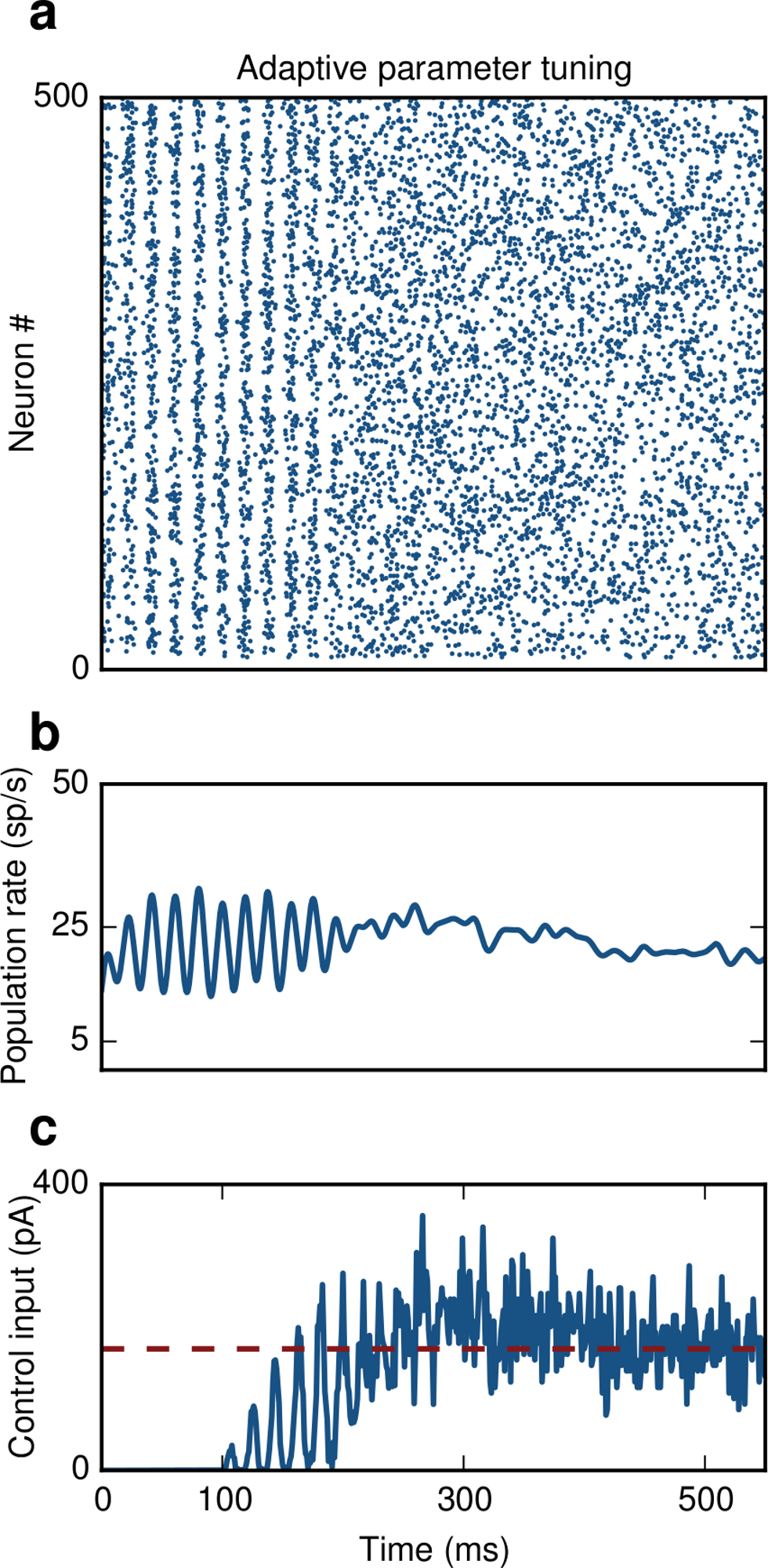

Supplement: S4 Fig — An adaptive procedure is used to find the optimal value for the control gain K. (a) I network. Switching on the controller at t = 100 ms results in suppression of oscillations. (b) Population activity of inhibitory neurons (blue). (c) The algorithm converges to an optimal value (red dashed line) for the control input within 200 ms after initiation of the procedure. (TIF) [file pcbi.1004720.s006.tif]
